# The Development and Validation of the Healthcare Professional Humanization Scale (HUMAS) for Nursing

**DOI:** 10.3390/ijerph16203999

**Published:** 2019-10-19

**Authors:** María del Carmen Pérez-Fuentes, Iván Herera-Peco, María del Mar Molero Jurado, Nieves Fátima Oropesa Ruiz, Diego Ayuso-Murillo, José Jesús Gázquez Linares

**Affiliations:** 1 Department of Psychology, Faculty of Psychology, University of Almería, 04120 Almería, Spain; foropesa@ual.es; 2 Department of Psychology, Faculty of Psychology, Universidad Politécnica y Artística del Paraguay, Asunción 1628, Paraguay; 3 Health Sciences Collegue, Alfonso X El Sabio University, 28691 Madrid, Spain; 4 Consejo General de Enfermería, 28023 Madrid, Spain; d.ayuso@consejogeneralenfermeria.org; 5 Department of Psychology, Universidad Autónoma de Chile, Providencia 7500000, Chile; jlinares@ual.es

**Keywords:** validation, scale, humanization, healthcare personnel, health

## Abstract

Introduction: The approach and use of the term “humanization” is very much present in healthcare. However, instruments for measuring the concept of the humanization of care are yet to be designed and developed. Objective: The main objective of this study was to evaluate and validate the Healthcare Professional Humanization Scale (HUMAS) for nursing professionals. Method: The sample was made up of 338 adults, who were nurses working at health centers and hospitals, and aged between 22 and 56. Results: The results of the analyses confirm that the Healthcare Professional Humanization Scale (HUMAS) has an adequate construct validity and reliability, and defines the humanization of care as a multidimensional construct, made up of five factors: Affection, Self-efficacy, Emotional understanding, Optimistic disposition and Sociability. Conclusions: The new HUMAS scale may be an easily administered and coded instrument for approaching the humanization of care, not only in research, but also in practice.

## 1. Introduction

The approach to and use of the term “humanization” is very much present in the area of care, and has even come to be defined as the humanization of care, arising in response to the perception of a society where healthcare is dehumanized or depersonalized [1,2,3,4]. This is a situation that, even though considered multifactorial [5], may be associated to a great extent with emotional exhaustion, burnout [6,7,8], stress [2] or other factors more closely associated with the workplace, such as staffing ratios or the automation of care [2,9]. 

However, in spite of its rapid introduction and acceptance in the healthcare environment, there is still no clear definition of the concept of the humanization of care, which makes it somewhat vague, and has caused it to be considered a passing fashion by many. In this sense, there are various approaches directed toward the definition of the humanization of care, such as the one that defines care centered on the patient (patient-centered care) [2,10,11,12], or person-concentrated care [2,4], which makes it extrapolatable to healthcare professionals themselves, in addition to patients [3,13,14]. However, it should not be forgotten that the humanization of care is also understood as providing services and technologies, human resources and materials, and infrastructure for offering attention and ensuring the comfort and wellbeing of health service users [15]. 

However, it seems that all the approaches coincide in always offering a definition based on responding to patient’s needs, e.g., the need to receive information, have face-to-face communication with the healthcare professionals, and even receive the coordination of care [16]. 

These needs, expressed by patients, mean that the humanization of care must focus upon offering respectful and responsible care with respect to patient preferences, needs and even their values [10], and the same formula must be applied to their families, which is very necessary, for example, in pediatrics [12], Intensive Care Units [17,18,19], emergencies [14,20] and so forth. 

### 1.1. Definition

The humanization of care, as such, is based on the interaction between various essential actors: (i) Patients and family members, (ii) healthcare professionals and (iii) managers [14]. However, obviously, when dealing with care offered to the patients, the key element, without which the humanization of care cannot be understood, is the involvement of healthcare professionals in charge of offering that care to patients [3,17]. 

Therefore, the humanization of care involves healthcare personnel present in a set of social, personal and emotional resources, which characterize their professional practice, facilitate their job performance and enable them to take an active role in the recovery process. It is an approach to a combined construction of health, where the professional is able to respect individuals and be sensitive to their needs, whilst also providing them with tools for holistically improving their health [12,19], based on inter-subjectivity and reciprocity. 

**Figure 1:**
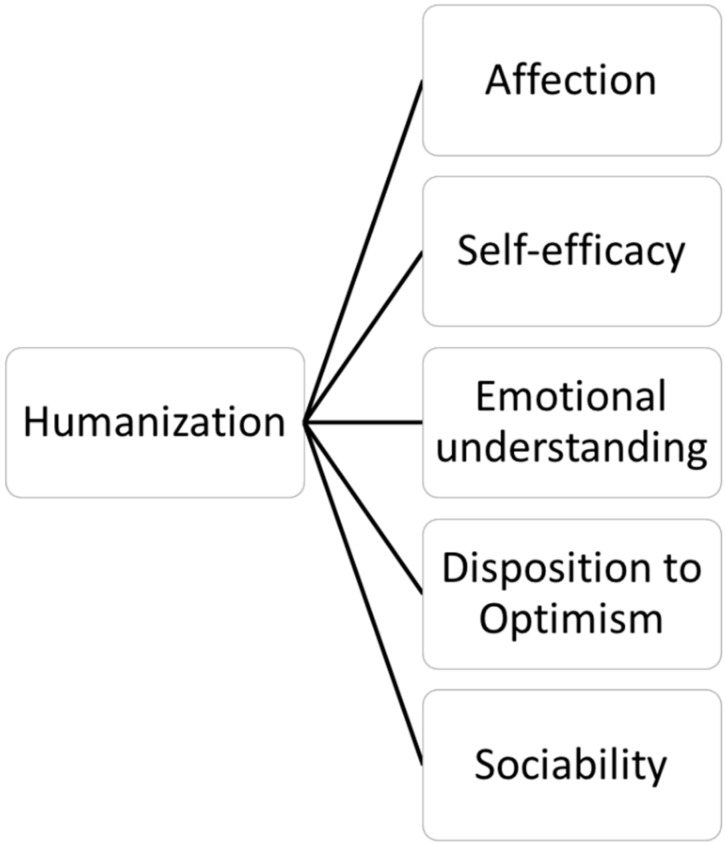
Explanatory theoretical model of the humanization construct in the Healthcare Professional Humanization Scale (HUMAS) Model.

The humanization construct is defined as a set of personal competencies that enable professional activity to be developed in the field of healthcare, respecting and watching out for the dignity and respect of the human being. It is therefore an activity focused on improving physical, mental and emotional healthcare, and is directed at both patients and healthcare professionals themselves, both groups being participants in the humanization of care. Thus, the model proposed (Figure 1) assembles all those attitudes that healthcare professionals must develop in order to incorporate humanization into their professional practice. 

### 1.2. Dimensions

If the main objective of the humanization of care is to offer the best possible care and satisfy patients’ needs, contact with healthcare professionals committed to that objective is indispensable. Some characteristics seem to be linked to it (Figure 1), for instance, it may be essential for these professionals to be committed to their work, which has already been demonstrated to achieve better results in patient care [21,22,23]. 

One element strongly associated with commitment to work, such as caregiving, is self-efficacy [23], understood as the healthcare professional’s skill in successfully managing complex and stressful situations [23,24]. Expectations concerning self-efficacy depend upon four factors: Achievements in work, vicarious and imaginary experiences, verbal persuasion and physiological and emotional states [25,26]. Their development begins in childhood and continues through adulthood. The development of symbolic thinking based on language development enables children to acquire a greater awareness of themselves and of their influence on their surroundings, promoted by the sensitivity of their main caregivers to their needs [25]. This skill is also closely related to self-esteem, which in turn is associated with the ability of individuals to become integrated and relate to people around them [27]. 

Moreover, some authors, such as Lown et al. [28], have mentioned the importance of developing empathy, the ability to socialize and work as a team [29], as an indispensable part of healthcare professional teams concerned with the development of person-centered care [28]. Sociability refers to the preference for seeking the company of other people. It is the ability to relate to others in an appropriate manner with assertiveness and empathy [30,31,32]. It has been demonstrated that there is an innate predisposition toward sociability, which evolves throughout childhood and causes maturity in certain brain areas and social interaction [33].

Another important factor associated with the commitment to the humanization of care is related to people’s ability to process emotional information in their work setting [34], and this skill is very important for finding job satisfaction, and therefore committing to a pursued objective [34,35]. In affection, one feels for another person, that is, one empathizes emotionally with the affective state of another person, but one’s own feelings are not confused with those of others [36,37]. Such processing of emotional information takes place in the limbic system of the brain [37,38]. From the first days of life, babies are affected when they hear other babies crying. This is considered the first antecedent of empathy [32]. Some authors think that cognitive and affective empathy should go together, and that there are different forms or levels of empathy [36].

Another of the skills associated with the humanization of care is active listening [5], understood as the social skill of understanding another’s point of view, as well as understanding and rationally identifying the emotions of those around you, expressing that knowledge and making recommendations that can reflect the expressed understanding [27,39,40]. This emotional understanding consists of cognitively empathizing with others, placing ourselves in their place, but without confusing our feelings with theirs [41], and it takes place in the prefrontal cortex of the brain [37,38]. Cognitive empathy and the capacity for mentalization are terms that have been used in the literature to refer to the same phenomenon, although there have been some attempts made at their differentiation [42]. On an evolutionary level, the capacity for mentalization is already manifest at three or four years of age and develops throughout life [43]. The adoption of a perspective evolves from the stage of egocentric thinking (3–6 years), until one is able to adopt the perspective of the other person (starting at 12 years of age) [44,45].

The humanization of care also involves an optimistic disposition, which generates positive future expectations and promotes coping with adversities and stressful situations in a professional practice [46]. Optimism has been associated with an increase in physical and psychological health, as well as with an improvement in social relations in general. It is a desirable characteristic that implies motivation [47,48]. Optimistic persons tend to be positive and concentrate their attention on favorable life events [30]. Optimism usually generates a feeling of self-confidence, which enables the internalization of success, motivating the person to achieve their own goals and relativizing negative events [49]. 

This way of perceiving and interpreting life events is learned in childhood. However, although children are usually extremely optimistic, they lose much of their optimism after puberty and in adulthood. Negative life events can convert optimism into pessimism [49], and in such circumstances, the role of personality variables must also be considered.

Nevertheless, and in spite of the importance of the humanization of care, which has even been included in national health programs, such as Brazil’s National Humanization Policy [13,15], or the Region of Madrid’s Health Care System Humanization Plan [17], there is no scientific bibliography, nor a validated instrument focusing on evaluating the humanization of the care concept itself, understood as the set of competencies that healthcare professionals, who are those who provide patient care, should have to effectively and humanely care for patients. 

Therefore, starting out from this conceptualization, as well as the main variables with which the humanization of care is related, and further, keeping in mind its relevance in different contexts, in a wide variety of healthcare professions, and in different areas, this study proposed, as its main objective, to evaluate and validate the Healthcare Professional Humanization Scale (HUMAS). We are convinced that the healthcare system must rotate toward a paradigm of thinking in which both professionals and patients take an active role in care, and place patients at the center of the healthcare system, in such a way that they participate in the management and care of their own health [50,51]. This means providing patients with educational and personal tools that enable them to cope with and progress in their health condition, so that they can offer quality care that centers on the individual, thus energizing interaction in care.

## 2. Materials and Methods 

### 2.1. Participants

The sample was made up of 338 nurses working at several different centers in Spain (Hospitals, Health Centers, etc.). They participated in the study by filling out a questionnaire voluntarily, when they received or found out about the study. The sample is therefore of nurses who were actively employed at the time that the data were collected. Incomplete questionnaires or questionnaires with random answers were discarded (detected by control questions: Seven eliminated by Control Question 1; and one by Control Question 3). Thus, the final sample consisted of a total of 330 subjects, of whom 63.9% (n = 211) were working on temporary contracts, and the remaining 36.1% (n = 119) had permanent contracts.

The mean age of the participants was 32.3 (SD = 7.55), with a range of 22 to 56. Of the total sample, 83.9% (n = 277) were women and 16.1% (n = 53) men, with a mean age of 30.62 (SD = 4.91) and 32.62 (SD = 7.92), respectively. 

### 2.2. Instruments

An ad hoc questionnaire was prepared to collect the sociodemographic data (age, sex, marital status and degree), as well as compiling information on their profession and job: Years of experience, employment situation (permanent or temporary), work shifts (rotating, 12 h or longer, nights only, morning/afternoon), and number of users attended to in a work day. 

The Healthcare Professional Humanization Scale (HUMAS). The humanization construct was defined as shown in the section on its definition and dimensions (see Figure 1). 

In this model, the accent is placed upon cognitive empathy, the dignification of the subject and the adaptation of healthcare spaces with a friendly, close decoration. Thus, healthcare professionals must not only have technical competencies related to their job, but also personal competencies that assist in creating a humanized professional job performance.

Basic Empathy Scale [52]. This is adapted from the brief version by Oliva et al. (2011) [53], for Spanish adolescents, based on the original Basic Empathy Scale designed by Jolliffe and Farrington (2006) [54]. It has nine items, which provide a score in Affective Empathy (feeling vicariously through the other person) (Items 1, 2, 3 and 6), a score in Cognitive Empathy (that is, “realization”) (Items 4, 5, 7, 8 and 9) and a total score in empathy. In this study, the Cronbach’s Alpha was α = 0.88, α = 0.86 for Affective Empathy and α = 0.91 for Cognitive Empathy.

Positivity Scale [55]. This scale measures positivity, defined as the tendency to see life and experiences with a positive perspective, through eight items, like “I have great faith in the future”. In general, the reliability and validity coefficients were optimal, with robust psychometric properties [55], and in this study, α = 0.83. 

Pro-sociability Scale [56]. This is adapted from the Pro-sociality Scale by Caprara et al. [57]. It is comprised of 10 items, which evaluate prosocial behavior based on two dimensions, prosocial behavior and empathy and emotional support, with α = 0.91 and α = 0.81, respectively, for this sample.

### 2.3. Procedure

A map of the construct was prepared, with the definition and the dimensions most frequently related to it in the literature as the starting point (Communication, Attention, Positivity, Empathy, Emotional Control, Communication skills, Emotional Intelligence, Optimum Experiences—FLOW, Ethical Sensitivity, Affection, Active Listening, etc.). A total of 167 items were distributed in different dimensions, derived from the revision of the scientific literature and associated with the humanization construct. Then, the Delphi technique was employed by a panel of experts, which led us to the elimination of 17 items considered unrelated to the concept, which were already evaluated in another dimension. Later, a pilot study was carried out with ten nursing professionals to detect items that included words that were unknown to them, or confusing expressions. 

Prior to collecting the data, compliance with information standards, confidentiality and ethics in data processing were guaranteed to the participants. The study was approved by the Bioethics Committee of the University of Almería (UALBIO2019/30). The General Nursing Council distributed the survey among its members through its Bulletin, e-mail, social networks, etc. Simple random sampling was used. The questionnaire was implemented on a Web platform that enabled participants to fill it out online. A series of control questions were included for the control of random or incongruent answers, and such cases were discarded from the study sample. 

### 2.4. Data Analysis 

This study is quantitative, observational and cross-sectional. Data analyses were performed in two stages, following the steps for validation recommended by Álvarez-García et al. (2017) [58] and Pérez-Fuentes et al. (2017) [24]. In the first stage, the HUMAS structure was studied. To approach this objective, various preliminary analyses were conducted (exploratory factor analysis), and later, a confirmatory factor analysis (CFA) of the HUMAS humanization model was proposed, taking the following fit indices as measures: χ2/df, Comparative Fit Index (CFI), Tucker-Lewis index (TLI), Root Mean Square Error of Approximation (RMSEA), with its confidence interval (CI) at 90%. The χ2/df was used, considering values below five [59], a CFI and IFI over or near 0.95, and an RMSEA below or very near to 0.06 to be acceptable [60]. As a general rule, the model had a good fit when: The 2/DF ratio ≤ 3; GFI, AGFI and TLI > 0.90; CFI > 0.95; and RMSEA ≤ 0.05. The appropriate re-specifications of the proposed model were made, considering theoretical and statistical criteria (change indices, errors of estimation, and standard error of measurement). The Akaike Information criterion (AIC) [61] was used for the model selection. 

The construct validity (Empathy, Pro-sociability and Positivity) was analyzed in the second stage, and an analysis supporting the invariance of the factor structure, proposed for each contract type (permanent/temporary), was provided. Tests of invariance enable valid inferences about differences in latent variables to be drawn for different populations, so the goodness of fit of these groups was tested separately (Models M0a-Permanent and Model M0b-Temporary). 

The resulting four nested models were evaluated: (a) Model 1: Configuration invariance (factor structures invariant across groups); (b) Model 2: Metric invariance (adding invariant factor loads); (c) Model 3: Scalar invariance (adding intercepts by invariant elements); and (d) Model 4: Strict invariance (adding invariant residuals), with no consensus criterion for determining the criteria to be employed in evaluating the difference in the fit of the nested models [62]. This study used the ΔCFI to evaluate the fit. Thus, the model was interpreted as completely invariant, if the value found by the ΔCFI was below 0.01 [63]. 

Finally, for the construct validity, the correlations between the scores of the different HUMAS factors and between the General Humanization Factor and the intrapersonal, interpersonal, stress management, adaptability, mood, affective and cognitive empathy, positivity, personal impact, job dissatisfaction, social climate and motivational quitting factors, were found.

The analyses were performed using the SPSS Statistical Package, version 23.0, for Windows and the AMOS 22 Program (IBM, Chicago, IL, USA). 

## 3. Results

### 3.1. Preliminary Analysis

First, the data show that the HUMAS item distribution is within the limits of normality, according to Finney and DiStefano [64], for whom 2 and 7 are the maximum values allowed for skewness and kurtosis, where the maximum values in our case were 1.46 and 2.26, respectively. The exploratory factor analysis used the extraction of the principal components with Varimax rotation (KMO = 0.88), based on the exploratory analysis of the theoretical model proposed for the humanization construct. 

**Figure 2:**
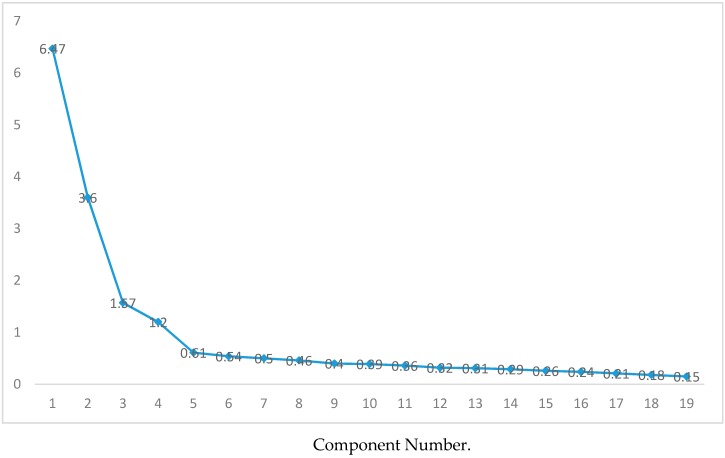
Scree plot for the factor analysis of the scale, following the proposed theoretical model.

The principal component analysis (chosen because the determinant level, *p* = 1.149, shows an inter-correlation of variables, which is a requirement for using this method) revealed the existence of five components with eigenvalues over 1. Thus, the Scree Plot shows the adequacy of the rotation with five factors, with eigenvalues from 6.47 to 1.12, since these factors are clearly distanced from the sixth, with a score of 0.61 (Figure 2). 

After the factor analysis, we selected the items with factor saturations over 0.50 from the matrix of rotated components (Varimax Rotation). Table 1 shows how Factor 1 corresponds to the items that include Affection in the scale. Factor 1 is composed of five items, all with a weight over 0.79, explaining 34.07% of the variance. Factor 2 is composed of five items that make up the Self-efficacy component and explain 18.93% of the variance. Factors 3, 4 and 5 are made up of three items, and explain 8.27%, 6.30%, and 5.90% of the variance, corresponding to the Emotional understanding, Optimistic disposition and Sociability components, respectively. 

### 3.2. Confirmatory Factor Analysis of the HUMAS Model

**Figure 3:**
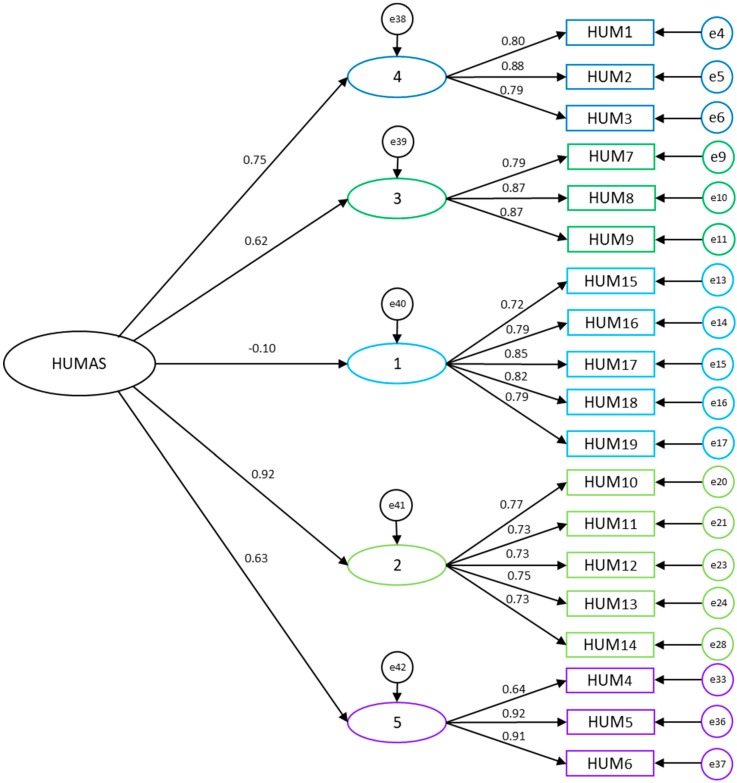
Proposed HUMAS Model with GF (n = 330). Note: F1: Affection; F2: Self-efficacy; F3: Emotional understanding; F4: Optimistic disposition; F5: Sociability.

Table 2 shows the fit of the different models of the scale: The HUMAS model proposed with and without the general humanization factor. It may be observed that the model without the general factor and the model with the general factor show adequate values. The HUMAS model with the General Factor (Figure 3), which consists of five factors and corresponds to what was found in the exploratory analysis and the theoretical proposal, fit best, once the appropriate re-specifications were made, considering the theoretical and statistical criteria (change indices, errors of estimation and standard errors of measurement). The difference between the AIC Default model = 368.830 and the AIC Saturated model = 418.000 is also observed to be lower than that between the other model, showing that this is probably the best model, according to the Akaike criteria for model selection. 

### 3.3. Construct Validity Analysis

The convergent validity analysis of the questionnaire (Table 3), with respect to the Empathy Scale, the Pro-sociability Scale and the Positivity Scale, show that practically all of the factors are positively correlated, except for affective empathy, which showed no relationship with the general humanization factor, and a negative relationship with the Affection factor. 

Table 4 shows the values for the six different models, where ΔCFI is over 0.01 in Models 1, 2 and 3, which is an acceptable configural, metric and scalar invariance. Specifically, the ΔCFI between Model 1 (base model group) and Model 4 is 0.019, so strict invariance cannot be accepted. 

## 4. Discussion

Several different focuses and studies have reported the need to seek a consensus with respect to the term, the humanization of care, which would contribute systematically to its development in the field of health [2]. In this respect, previous studies have agreed that the humanization of care is built upon people’s needs in the care relationship [4,10,11,16], for which purpose healthcare professionals must be committed to their work [17,21,22,23], which is promoted if they possess certain characteristics [27,28,29,34,39,40,46].

This study met its main objective of evaluating and validating the humanization scale in healthcare professionals (HUMAS). With respect to the concrete dimensions that HUMAS contributes, the exploratory and confirmatory factor analyses performed revealed the existence of five clear and coherent factors with regard to its content and psychometric properties: Affection (processing emotional information adequately and empathizing with the affective state of another person), Self-efficacy (confidence in one’s own actions in managing complicated and stressful situations successfully), Emotional understanding (understanding, identifying and rationally interpreting the feelings and emotions of other people), Optimistic disposition (positive expectations about future events) and Sociability (preference for seeking the company of other people, ability to relate to others in an appropriate manner, with assertiveness and empathy). 

Concerning the performed analyses, with the five main factors extracted from HUMAS, statistically significant positive correlations were found between the five factors and the empathy scales (the ability to put oneself in another’s place, interpreting and understanding what is happening to them, with a healthy personal identification with their emotions), pro-sociability (understanding those behaviors, tending to help others) and positivity (tendency to see life and experiences with a positive perspective), except in the case of affective empathy, which did not correlate with the general humanization factor, and was negatively associated with Affection. Thus, the higher the scores in Self-efficiency, Emotional understanding, Optimistic disposition and Sociability, the greater were the Empathy, Pro-sociability and Positivity of the nursing staff. Concerning the relationship between the Affection Factor and Empathy, the higher nurse scores were in Affection, and the lower ones were in affective empathy. Studies have emphasized the role of emotional information processing and active listening in job commitment and humanization [5,27,34,39,40], and like self-efficacy, which is closely related to self-esteem, it contributes, along with openness to sociability, to the humanization of care in the healthcare environment [23,27]. These personal, emotional and social factors are reinforced if the healthcare professionals show a cognitive optimistic disposition, which enables them to distance themselves slightly from pain, be less affected by illness [47] and improve their cognitive empathy, which facilitates taking the perspective of and understanding the mental state of another person [27], further enabling more optimal coping with adversities and stressful situations, associated with professional job performance in healthcare [46]. 

The new HUMAS scale may be an easily administered and coded instrument for approaching the humanization of care in the context of health, based on five fundamental dimensions. Even so, when generalizing the results, it should be borne in mind that the sample is specific to the field of nursing, which is one of the limitations of this study. Similarly, it deals with a small sample of nurses with a larger number of women than men, and although this does reflect the characteristics of the population, it should still be taken into account in interpreting the results.

## 5. Conclusions

The results of the analyses confirm that the Healthcare Professional Humanization Scale (HUMAS) has an adequate construct validity and reliability, defining the humanization of care as a multidimensional construct made up of five factors: Affection, Self-efficacy, Emotional understanding, Optimistic disposition and Sociability.

As futures lines of research, we should apply this model and instrument to other healthcare professionals (e.g., doctors, physiotherapists, etc.), and also in specific contexts (e.g., intensive care, the emergency room, etc.). Furthermore, the influence of affective empathy on the level of humanization shown by healthcare professionals could be studied, as it shows a negative relationship with some of the HUMAS factors, such as affection, and does not relate to the general humanization factor in the scale, which could determine the ideal limits that ensure that affection and empathy go together, exploring the role of the personality factors in this sense.

Finally, the construction of this scale may contribute to the development of research on the humanization of care in health professionals (nurses, doctors, nursing aides, psychologists, social workers, etc.) and in different contexts. Thus, the psychometric indicators, both for the factors and for the global scale, reveal that it is a valid, reliable measurement instrument for use, not only in research, but also in practice. This instrument will provide a measurement of the benefits of intervention programs directed at implementing actions based upon humanized care. It will also enable us to find out whether the units or centers with the highest level of HUMAS have the best results in terms of patients’ evaluation of their care quality. 

## Figures and Tables

**Table 1:** Factor structure, communalities (h2), eigenvalues, Cronbach’s alpha and percentage of the explained variance (n = 330). Extraction method: Principal Component Analysis (PCA).

	F1	F2	F3	F4	F5	*h* ^2^
Item1				0.830		0.782
Item2				0.820		0.818
Item3				0.753		0.728
Item4					0.702	0.627
Item5					0.878	0.871
Item6					0.858	0.859
Item7			0.819			0.770
Item8			0.858			0.833
Item9			0.856			0.826
Item10		0.772				0.694
Item11		0.645				0.602
Item12		0.713				0.642
Item13		0.761				0.683
Item14		0.752				0.662
Item15	0.791					0.637
Item16	0.836					0.717
Item17	0.870					0.763
Item18	0.849					0.739
Item19	0.835					0.710
Eigenvalue	6.47	3.59	1.57	1.19	1.12	
Percentage of explained variance	34.07	18.93	8.27	6.30	5.90	73.47
Kaiser-Meyer-Olkin	0.88					
Bartlett’s sphericity	*χ*^2^_(171)_ = 3660.68, *p* < 0.000					
Cronbach’s alpha	0.89	0.86	0.88	0.86	0.85	0.86

Note: The items are listed in decreasing order of saturation. Visualization coefficient > 0.50. F1: Affection; F2: Self-efficacy; F3: Emotional understanding; F4: Optimistic disposition; F5: Sociability.

**Table 2:** Fit indices for the proposed models (calibration sample: n = 330).

Model	*χ*^2^ (*df*)	*χ*^2^/*df*	CFI	TLI	IFI	RMSEA		
						Est.	CI90%	
							Low	High.
HUMAS Model without GF	225.289 (142)	1.586	0.977	0.972	0.977	0.042	0.031	0.052
HUMAS Model with GF	244.830 (147)	1.665	0.973	0.968	0.973	0.045	0.035	0.055

Note. CFI = Comparative fit index; TLI = Tucker-Lewis index; RMSEA = Root Mean Square Error of Approximation; CI = Confidence Interval; df = Degrees of freedom; Est. = Estimation.

**Table 3:** Correlations. Convergent analysis of HUMAS.

	1	2	3	4	5	6	7	8	9	10
1. Affective Empathy	-									
2. Cognitive Empathy	0.420 **	-								
3. Prosocial Behavior	0.283 **	0.557 **	-							
4. Empathy and Emotional Support	0.395 **	0.579 **	0.761 **	-						
5. Positiveness	0.042	0.397 **	0.510 **	0.408 **	-					
6. Optimistic disposition	0.015	0.258 **	0.342 **	0.282 **	0.668 **	-				
7. Sociability	0.242 **	0.398 **	0.557 **	0.472 **	0.421 **	0.493 **	-			
8. Emotional understanding	0.053	0.300 **	0.337 **	0.407 **	0.359 **	0.422 **	0.310 **	-		
9. Self-efficacy	0.107	0.434 **	0.630 **	0.577 **	0.609 **	0.577 **	0.533 **	0.522 **	-	
10. Affection	−0.131 *	0.140 *	0.218 **	0.053	0.311 **	0.114 *	0.149 **	−0.068	0.067	-
HUMAS	0.024	0.432 **	0.599 **	0.479 **	0.695 **	0.676 **	0.634 **	0.555 **	0.734 **	0.615 **

**. Correlation is significant at the 0.01 level (two-tailed). *. Correlation is significant at the 0.05 level (two-tailed).

**Table 4:** Multi-group analysis of invariance by the type of contract (permanent/temporary).

Model	χ^2^	df	χ^2^/df	Δχ^2^	CFI	ΔCFI	IFI	RMSEA (IC 90%)
M0a (permanent)	418.270 (*p* = 0.000)	294	1.423		0.966		0.966	0.036 (0.028–0.044)
M0b (temporary)	418.270 (*p* = 0.000)	294	1.423		0.966		0.966	0.036 (0.028–0.044)
M1 (base model groups)	418.270 (*p* = 0.000)	294	1.423		0.966		0.966	0.036 (0.028–0.044)
M2 (SF)	429.385 (*p* = 0.000)	308	1.394	0.029	0.966	-	0.967	0.035 (0.026–0.042)
M3 (SF + Int)	463.959 (*p* = 0.000)	327	1.418	0.005	0.962	0.004	0.962	0.036 (0.028–0.043)
M4 (SF + Int + Err)	546.904 (*p* = 0.000)	356	1.536	0.113	0.947	0.019	0.947	0.040 (0.034–0.047)

Note: FS = Factor Saturations, Int = Intercepts, Err = Errors.
